# Epidemic amidst the coronavirus disease-19 pandemic

**DOI:** 10.7189/jogh.11.03056

**Published:** 2021-06-05

**Authors:** Rohan Kumar Ochani, Farah Yasmin, Nadia Nazir Jatoi

**Affiliations:** Department of Internal Medicine, Dow University of Health Sciences, Karachi, Pakistan

Coronavirus disease-2019 (COVID-19) pandemic caused by the severe acute respiratory distress syndrome coronavirus-2 (SARS-CoV-2) initially originated in the city of Wuhan, China. Due to the high infectivity rate of the virus, it rapidly spread across international borders affecting over 200 countries and territories worldwide. It has resulted in significant mortality consuming more than half-a-million lives, as of July 2020 [1]. Since then, research scientists across the world have been engaged in collaborations with health care authorities and pharmaceutical companies to devise an efficient vaccine and effective treatment options to curtail the pandemic [2].

However, whilst scientists and medical professionals globally have been involved in diagnosing and managing the number of increasing COVID-19 cases, they are now alarmingly concerned about any potential outbreaks that might arise bearing the risk of epidemics having the potential to significantly impact the already overburdened health care systems around the world. This would further collapse the already overburdened health care system, tremendously affect the economy and result in loss of lives.

However, this situation is practically very unlikely to not happen. Recently in July 2020, the Chinese public health authorities issued a standard bubonic plague outbreak alert following which the local authorities issued a citywide level 3 warning for plague prevention, the second lowest in a four-level system. A confirmed case of bubonic plague was diagnosed in a herder in the city of Bayannur, Inner Mongolia Autonomous Region, located northwest of Beijing, China. Previously, cases of bubonic plague have also been reported in western Mongolia due to the consumption of infected marmot [3]. According to the World Health Organization (WHO), the bubonic plague is one of the three infectious diseases plague caused by the bacterium Yersinia pestis, the other two being the septicaemic plague and pneumonic plague. These zoonotic bacteria are usually found in small mammals and fleas and, therefore, can easily be transmitted from animals to humans by the bite of infected fleas, direct contact with infected tissues, and inhalation of infected respiratory droplets [4]. Consequently, the officials at Inner Mongolia’s regional center for disease control have been alerted about the risk of human-to-human transmission and long-distance transmission leading to an epidemic and thus have banned hunting, skinning, and consumption of infected animals [3].

The main clinical manifestations of the bubonic plague comprise of fever, headache, chills, weakness, and painful lymph nodes. The bubonic plague multiplies in the lymph nodes and accordingly results in the painful swelling of the lymph nodes also known as ‘buboes’ [4]. The bubonic plague carries a high mortality risk with a case-fatality ratio of 30% to 60% and has previously resulted in the ‘Black Death Pandemic’ claiming more than 50 million deaths in Europe in the middle ages. However, with the advent of modern medicine, it can now be managed with antibiotics, and therefore, early diagnosis and treatment are essential for the reduction of complications [3]. However, due to human-to-human transmission, especially in developing countries where not everyone has access to antibiotics, many might become more affected by this plague. This calls for clinicians to remain vigilant for any suspected cases of bubonic plague and conduct on-going surveillance to prevent the risk of an epidemic in the Inner Mongolia Region.

**Figure:**
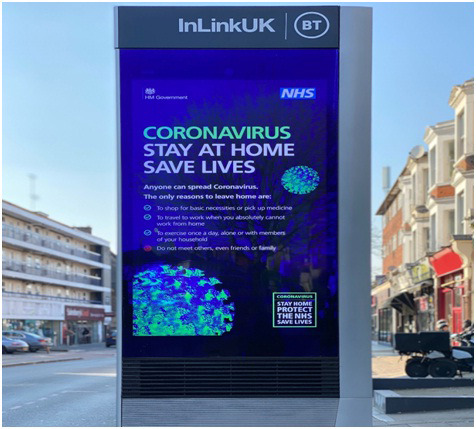
Photo: Increasing awareness amongst the public for precautionary measures might help in reducing transmissibility of other infectious diseases (from the author’s own collection, used with permission).

Cases of bubonic plague have also been reported in other countries apart from China. According to the WHO, nearly 1000-2000 people get affected by the plague annually. The possibility of plague exists in almost every continent such as the western United States (US), southeast Africa, India, and the Middle East. Furthermore, a few cases of plague get reported every year in the US, for instance, eight plague cases have been previously reported in Colorado in 2014 [3]. These cases usually go unnoticed by the public who are busy with their everyday lives. However, amidst the COVID-19 pandemic, people are confined to their homes due to lockdown of businesses making them confined and feeling lonely, many of whom utilize social media to keep a constant check on critical updates regarding the current pandemic and news of any potential infectious disease outbreaks such as these plague cases might lead to increased hysteria among the public, and hence increase the probability of a mental health epidemic in different regions of the world following the COVID-19 pandemic.

These emerging cases have already created fear, anxiety, and worry amongst the public and any new epidemics would further increase the prevalence of psychological issues in today’s world, particularly anxiety and depression. Nearly half (49%) of the US residents showed signs of depression ranging from moderate to severe while 3 out of 5 US residents remained scared of contracting the SARS-CoV-2 [5]. While it is essential for the public to not panic about these recent plague cases, however, the scientists and physicians must be on the lookout for any novel epidemics and must remain alert to keep new ones at bay.

The adoption of efficient self-protection measures such as the common social-distancing adopted by the public during the COVID-19 pandemic will also likely reduce the risk of other communicable diseases such as the seasonal influenza infection. For example, in China alone, the cases, according to a July 2020 report, of influenza had decreased from 29 000 a month to 23 000 after imposing of lockdown [6]. Even though there is a daunting threat of epidemics, the public might be better prepared to protect themselves due to the pandemic since they are vigilant and quick to report abnormal health conditions promptly to their health care physicians which might lead to efficient recognition of pathogens and halt its spread immediately. Nevertheless, efforts to minimize the panic amongst the public is essential for the well-being of society.

Additionally, the bubonic plague can be prevented by antibiotics that might lead to the over-buying of antibiotics. Antibiotics can also be sold at increased prices since the drug-makers were previously reported to hike their drug prices during the COVID-19 crisis, increasing prices on average 23.8 percent for 245 drugs, many of which are used in COVID-19 treatments or research [7]. This added financial burden will indefinitely make the population more anxious and therefore may add to the psychological impact that the existing pandemic poses. Moreover, according to the recently published reports, the COVID-19 pandemic has accelerated the threat of a global antimicrobial resistance since COVID-19 patients admitted to the hospital and even those with mild to moderate symptoms receive prophylactic antibiotics to minimize the risk of secondary bacterial infections such as bacterial pneumonia. This would cause the bacteria to mutate and reproduce giving rise to colonies that are now resistant to antibiotics [8]. This implies that this antibiotic resistance would make it difficult to manage any newly arising bacterial epidemics.

Regardless of the emerging threats of the epidemic, it is worthy to mention that the public health care sector might be better prepared than ever before, due to the pandemic. The COVID-19 pandemic has led better policies and methods being implemented all over the world to prevent the transmission, which also includes any unknown pathogens. For instance, in China, extra funds were allocated for public health, prevention, and management of any epidemics, and well-equipped shelter hospitals were built. In Italy, according to the response plan, more intensive care units (ICUs) were set up, the algorithm to detect affected individuals were made, and training of the staff instituted [9]. These efforts will promptly prepare the countries and the health care systems for any emerging epidemic threat and the response would be much faster. Authorities like WHO will likely be more focused on public health to efficiently control infectious diseases as the emergence of SARS-CoV-2 after SARS-CoV in 2003, raises concerns regarding future events [10]. Moreover, the issue regarding the antibiotic resistance can also be efficiently solved now with antimicrobial peptide-antibiotics that minimizes the risk of antibacterial resistance [11]. The mechanism of action by which they achieve this goal lies in their rapid death kinetics and broad action as they are combination therapies. Moreover, they act on more than one target which reduces the chances of resistance exceptionally [11]. One other technique to minimize resistance is the novel nano-medicine which plays a significant role in increasing the effectiveness of existing drugs, by enhancing their physicochemical properties and stability, making it possible for biofilm internalization, prolonging antibiotic release, and capability of delivery to the site of infection [12]. Although these methods have been already highlighted before the pandemic, authorities are now increasingly alert since it was also observed by WHO that collaborative research and knowledge sharing across borders has helped to a great degree during the pandemic [13], hence the importance of immediate research to stop the spread is now being appreciated [13]. The prior preparedness of research centers and pooled experiences of the current pandemic in addition to witnessing effective measures such as social distancing helps in devising preemptive efforts for the future. According to the WHO advice on response to pandemic, countries, governments, and private companies have all come together to increase the resources and capacities of the health care system, hospitals, and the public health sector [12]. This allows authorities and respective countries to quickly tackle any likely new pathogen or infectious diseases we may encounter in the future.

Nevertheless, continued efforts are still needed to make sure emerging threats are minimized whilst the world faces a pandemic. We must not ever overlook the threat of any infectious disease emerging during or after the pandemic. In this regard, the media industry could play a prominent role in providing the public with authentic information concerning required precautions and treatment available such as antibiotics for the bubonic plague thus reducing public confusion. Perhaps, the government could establish a call-center comprising of a team of health care physicians responsible for not only providing authentic updates regarding the COVID-19 pandemic but also any potential infectious outbreaks or epidemics that would reduce the public anxiety and thus psychological burden.
